# Simultaneous Quantitative Detection of Helicobacter Pylori Based on a Rapid and Sensitive Testing Platform using Quantum Dots-Labeled Immunochromatiographic Test Strips

**DOI:** 10.1186/s11671-016-1254-7

**Published:** 2016-02-03

**Authors:** Yu Zheng, Kan Wang, Jingjing Zhang, Weijian Qin, Xinyu Yan, Guangxia Shen, Guo Gao, Fei Pan, Daxiang Cui

**Affiliations:** Institute of Nano Biomedical and Engineering, Department of Instrument Science and Engineering, Key Laboratory for Thin Film and Microfabrication of Ministry of Education, School of Electronic Information and Electrical Engineering, Shanghai Jiao Tong University, 800 Dongchuan Road, Shanghai, 200240 People’s Republic of China; The Outpatient Department of Zhujiang Hosptial, South Medical University, 1023 South Shatai Road, Guangzhou, 510515 People’s Republic of China

**Keywords:** Test strip reader, CMOS, Wi-Fi transmission, Kmeans algorithm, Urea enzymes, QD test strips

## Abstract

Quantum dots-labeled urea-enzyme antibody-based rapid immunochromatographic test strips have been developed as quantitative fluorescence point-of-care tests (POCTs) to detect helicobacter pylori. Presented in this study is a new test strip reader designed to run on tablet personal computers (PCs), which is portable for outdoor detection even without an alternating current (AC) power supply. A Wi-Fi module was integrated into the reader to improve its portability. Patient information was loaded by a barcode scanner, and an application designed to run on tablet PCs was developed to handle the acquired images. A vision algorithm called Kmeans was used for picture processing. Different concentrations of various human blood samples were tested to evaluate the stability and accuracy of the fabricated device. Results demonstrate that the reader can provide an easy, rapid, simultaneous, quantitative detection for helicobacter pylori. The proposed test strip reader has a lighter weight than existing detection readers, and it can run for long durations without an AC power supply, thus verifying that it possesses advantages for outdoor detection. Given its fast detection speed and high accuracy, the proposed reader combined with quantum dots-labeled test strips is suitable for POCTs and owns great potential in applications such as screening patients with infection of helicobacter pylori, etc. in near future.

## Background

Gastric carcinoma is a malignant tumor that is highly common all over the world and ranks second among all malignant tumors in China [1]. The cure rate of early-stage gastric carcinoma reaches up to 85–90 %, whereas that of late stage is less than 24 %. Thus, the early detection of gastric carcinoma is paramount to reduce the risk of patient mortality. Nowadays, along with the improvements to their standard of living, individuals have more concerned regarding health issues. Although modern medical technology can treat most diseases, patients suffer heavily during the treatment process. Nonetheless, researchers are still facing challenges in finding the cure for fatal diseases, such as cancer and HIV. Most diseases present high cure rates only when detected early. Therefore, early diagnosis is essential to reduce morbidity and mortality. In addition, early diagnosis of infectious diseases can prevent their development into epidemics. Helicobacter pylori are reportedly important microorganism that can be detected in human stomach. According to the data displayed, the mortality of gastric carcinoma is proportional to the infection rate of helicobacter pylori. More than half of people around the world had been infected with helicobacter pylori. As a result, the initiation and development of gastric carcinomas are closely linked to helicobacter pylori, which are important source of high-activity urea enzymes in the stomach [2–4]. Thus, urea-enzyme detection in patient serum samples by immunochromatographic assay can confirm early-stage gastric carcinoma.

Lateral flow immunochromatographic assay (LFIA) is a simple method for early detection [5–7]. This technique boasts of rapid detection rates, stable results, and cost-effective features. As such, LFIA is now widely used in the qualitative and semi-quantitative detection of several biomarkers, including antibodies, antigens, and even the products of nucleic-acid-amplification tests.

However, most of the results of lateral flow tests are assessed through visual observation. To obtain the numerical value of test outcomes, the operator must compare the result with a standard colorimetric card. Inevitably, individual differences in color discrimination render this value subjective and inaccurate. When the value is near the *cutoff* value, different operators may have conflicting judgments on the same results. To overcome this problem, many groups have developed matching test strip readers that can quantitatively determine LFIA values [8–15]. Mei et al. developed an embedded system based on an Acorn Risc Machine (ARM) processor to read a test strip [13]. However, the heavy weight of this device limited its application for outdoor detection. The majority of current strip readers are designed to run on desktop computers or laptops, which possess rapid processing speeds and stable performances [14, 15]. However, outdoor detection requires long durations of operation, but a laptop can work for only 2 or 3 h without a power supply. Existing strip readers are excessively large for portability. Moreover, images captured by image sensors are usually transmitted through a data cable, which is easily damaged when exposed to air, resulting in images with poor quality.

In this study, a complementary-metal-oxide-semiconductor transistor (CMOS) was used as the image sensor. The tablet personal computer (PC) was selected as the image-processing module for its longer battery life and lighter weight as compared to that of laptops. A Wi-Fi module was integrated into the strip reader to realize wireless image transmission and, consequently, improved image quality. In addition, an application compatible with the tablet PC’s hardware system was designed to process the image acquired by the CMOS sensor. The size of this reader was 230 mm × 146 mm × 75 mm, and the weight was approximately 2.5 kg. Subsequently, 100 positive and 100 negative urea-enzyme samples were used to evaluate our reader. The specificity and sensitivity of the reader were 97 and 95 %, respectively, thereby proving the stability and accuracy of our device.

## Methods

### Composition of the Test Strip

**Fig. 1:**
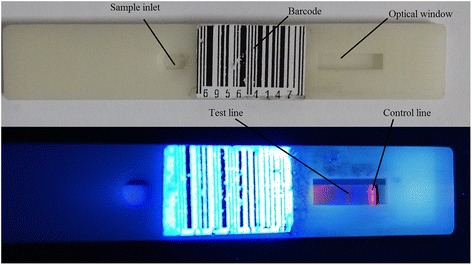
Test strip using a 3D printer

Water-soluble quantum dots (excitation and emission wavelengths were 365 and 620 nm, respectively) were prepared by our group’s member [16–18]. The antigen and antibody of the urea enzyme were both purchased from the Abcam (Shanghai, China). A standard immunochromatographic test strip typically consists of five parts, namely, sample pad, conjugation pad, nitrocellulose membrane, absorption pad, and polyvinyl chloride (PVC) backing. All the pretreated parts were assembled sequentially on a PVC backing card with 2-mm overlap of each component, as shown in Fig. 1. Antibodies were coated separately to serve as T-line and C-line. Both antibodies were dispensed by XYZ dispensing system to NC membrane. The quantum dot (QD) probe was dispensed onto the conjugate pad. After being dried at 37 °C, the assembly was cut into 3-mm-wide individual strip and then stored at room temperature inside a sealed plastic bucket with a desiccant until used. Bio-reagents are often immobilized in specific positions of the strip. When a liquid sample (such as human blood, saliva, or urine) is added onto the sample pad, the sample will flow from this component to the absorption pad on the membrane surface by capillary action. Once the sample reaches specific positions, the antigen in the sample will react with the conjugates (labeled with CdSe quantum dots). The residual sample continues to move forward and be absorbed in the absorption pad. After the reaction is completed, two lines will appear on the strip: the C-line (control line to confirm whether the strip is valid) and the T-line (test line used to judge the detection results). In this study, all of the test strips were prepared by our group [14].

Because the nitrocellulose membrane in the strip was very thin and fragile, the test strip was usually needed to be inserted into a cartridge. The LFIA cartridge was designed using Solidworks 2013 (Solidworks Corporation, Concord, MA, USA) and was then stereo lithographically printed using a 3D printer. The cartridge (Fig. 1) is primarily made of photosensitive resin, which exhibits good flexibility and adaptive hardness, and its precision reaches up to 0.1 mm. A sample slot is present on one end of the cartridge through which the liquid sample is to be added. Located in the middle of the cartridge is an optical window for signal acquisition. A barcode between these locations contains the patient information and the detection type.

### Design of Reader Hardware System

**Fig. 2:**
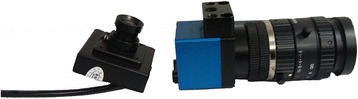
CMOS sensor (*left*) and CCD sensor (*right*)

**Fig. 3:**
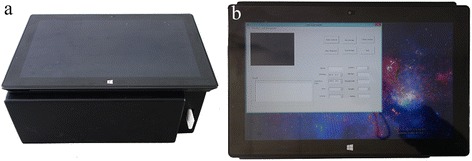
**a** Appearance of our test strip reader. **b** Application running on a tablet PC

A CMOS camera (Fig. 2, left side) was used as the image sensor in our reader because of the stable image quality it produces and its cost-effective price [19]. To illuminate the strip, a violet light-emitting diode (LED) was selected as the light source. A barcode scanner was utilized to read the barcode. As mentioned earlier, a tablet PC, owing to its light weight and stable performance, was utilized as the image processor (Fig. 3). The chosen tablet PC is lighter and thinner than a laptop. Designed by Microsoft company called Surface Pro, the tablet PC had a weight of 903 g and thickness of 13.5 mm. It has a fast processing speed and a small volume. More importantly, the tablet PC can run for 6–8 h without external power supply. These features validate the suitability of the selected device for outdoor testing. In addition, a Wi-Fi module was integrated into our reader. Whenever the camera captures an image, the Wi-Fi module would transmit the image to the tablet PC by wireless transmission. The information provided by the barcode scanner is also transmitted by wireless means. The Wi-Fi module can improve the integration of our reader as well as increase its stability and portability.

**Fig. 4:**
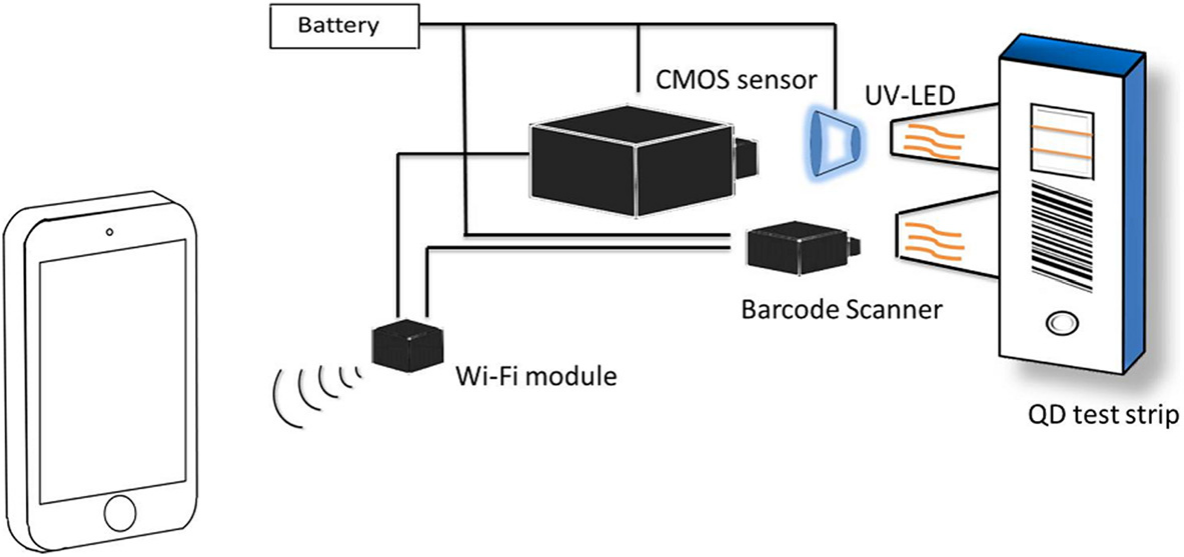
Hardware structure of the strip reader

The shell of our device was designed using the Auto Computer-Aided Design (AutoCAD) software. The hardware structure of our strip reader is shown in Fig. 4. The shell is made of engineering plastic, which has a light weight and good corrosion resistance. One side of the shell holds a slot for test strip insertion. Black was chosen as the color of the shell to attain a dirt-free appearance.

### Development of Reader Software System

In addition to the hardware system of our strip reader, a software system was designed to analyze the image and provide users with easy access to our device. The C++ programming language was employed in the design of the tablet PC application (Fig. 3b). The application was linked to a database, thus giving users the capability to save or load strip images and patient information.

The application opens with a log-in screen wherein users are asked to indicate their identities (i.e., doctors or patients). Doctors are given the highest authority in the application, whereas patients can only use the data query function. In the application’s main interface, the button labeled as “Image Acquisition” activates the camera. After image acquisition, the button labeled as “Automatic Detection” can be selected to calculate the fluorescence intensity of the T-line and the C-line. The screen displays a graph reflecting the trend of the fluorescence intensity, giving users an intuitionistic view. Then, the value and the result (i.e., positive or negative) are then displayed on the screen. The image can be saved on the hard disk for future comparison. As well, patient information is stored on the database with the corresponding image. The image is loaded along with the respective patient information.

The most useful information on the test strip is that of the T-line and the C-line. Background noise is usually present in the image captured by the camera. Thus, both lines warrant extraction from the raw image. We summed the fluorescence intensities of all the pixels in every column of the image. This summation allows for the detection of the strip edges. A cluster algorithm called Kmeans was used to calculate the average fluorescence intensity of the two lines [20–22]. First, a simple algorithm was applied to denoise the raw image [23]. Then, the acquired image was converted from the red-green-blue (RGB) format to the hue-saturation-intensity (HIS) format.

Given that an image contains *N* pixels, which will be allocated to the *N*_*c*_ cluster centers, the *i*th pixel to be allocated is designated as *V*_*i*_ and the *j*th cluster center is designated as *C*_*j*_. All pixels will be allocated to the nearest cluster according to the Euclidean distance. The location of the clustering center was randomly initialized. The procedure of the proposed algorithm is as follows.

1. All clustering centers should be initialized. We set the parameters *α*_*a*_ = *α*_*b*_ = *α*_0_ (*α*_0_ a constant ranging from 0 to 1/3). These parameters are used to control the fitness between *C*_*s*_ and *C*_*l*_.
2. We allocate all pixels to different clusters. We then recalculate the locations of all clustering centers according to formula 1 as follows:
  1 $$ {C}_j=\frac{1}{n_j}{\displaystyle {\sum}_{i\in {C}_j}\kern0.5em }{V}_i $$
  where *n*_*j*_ is the number of pixels in the *j*th cluster.
3. We calculate the fitness of every cluster using formula 2 as shown below:
  2 $$ \mathrm{f}\left({C}_j\right)={\displaystyle {\sum}_{i\in {C}_j}}{\left(\left\Vert {V}_i-{C}_j\right\Vert \right)}^2 $$
4. We find the minimum and maximum values of the fitness *f*(*C*_*j*_) and allocate *C*_*j*_ to *C*_*s*_ and *C*_*l*_.
5. If *f*(*C*_*s*_) < *α*_*a*_ ⋅ *f*(*C*_*l*_), *V*_*i*_ ∈ *C*_*l*_ and *V*_*i*_ < *C*_*j*_, *V*_*i*_ should be allocated to *C*_*s*_. Then, we recalculate the value of *C*_*s*_ and *C*_*l*_ according to formulas 3 and 4 as follows:
  3 $$ {C}_s=\frac{1}{n_s}{\displaystyle {\sum}_{i\in {C}_s}\kern0.5em }{V}_i $$
  4 $$ {C}_l=\frac{1}{n_l}{\displaystyle {\sum}_{i\in {C}_l}\kern0.5em }{V}_i $$
6. We recalculate the value of *α*_*a*_ according to the formula *α*_*a*_ = *α*_*a*_ − *α*_*a*_/*n*_*c*_. Steps 4 and 5 are repeated until *f*(*C*_*s*_) > *α*_*a*_ ⋅ *f*(*C*_*l*_).
7. We reallocate all pixels to the nearest cluster and recalculate the location of every clustering center according to formula 1.
8. We recalculate the value of *α*_*a*_ and *α*_*b*_ according to the formula *α*_*a*_ = *α*_0_, *α*_*b*_ = *α*_*b*_ − *α*_*b*_/*n*_*c*_. Steps 3 to 7 are repeated until *f*(*C*_*s*_) > *α*_*b*_ ⋅ *f*(*C*_*l*_)

### Clinical Specimen Examination

This study was approved by the Medical Ethics Committee of Shanghai Jiao Tong University. All the clinical samples were collected from Renjin Hospital, the affiliated hospital of Shanghai Jiao Tong University. A total of 200 urea-enzymes samples were tested using a reader system employing lateral flow strips labeled with quantum dots.

### Data Analysis

All data are presented in this paper as mean ± standard deviation. Statistical differences were evaluated using the *t* test and considered significant at *P* < 0.05.

## Results and Discussion

### Characterization of CdSe QDs

**Fig. 5:**
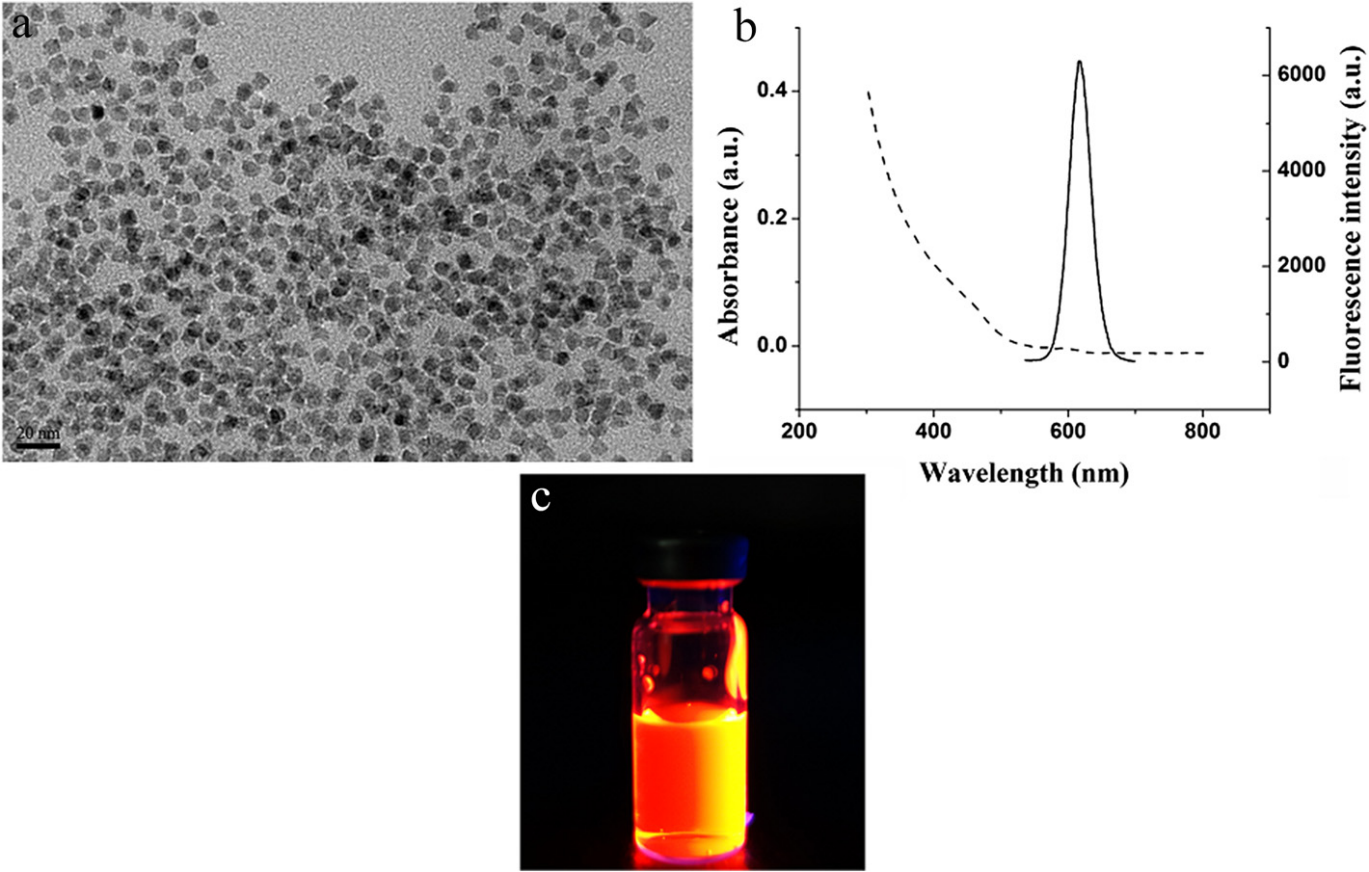
**a** TEM picture of synthesized CdSe QDs. **b** Absorption and emission spectrogram. **c** Digital photo of the QD-labeled urea-enzyme antibody after UV condition

The QDs were CdSe core/ZnS shell QDs with carboxy1 groups displayed on their surface, which formed 6-nm particles in diameter and had a good dispersion. The TEM picture of the QDs is displayed in Fig. 5a, showing that the water-soluble QDs have a diameter of 6 nm. The absorption and emission spectrogram of the urea-enzyme QDs was shown in Fig. 5b. The emission wavelength was approximately 620 nm. The digital photo of the QD-labeled urea-enzyme antibody after UV condition is shown in Fig. 5c.

### Reader Hardware System

Our group previously utilized a charge-coupled device (CCD) (Fig. 2, right side) as the image sensor because of the stable image quality it produces [15]. However, CCDs are expensive, which would result in the high cost of developing a strip reader from such a device. Moreover, CCD sensors require a data acquisition (DAQ) card to accomplish image capturing. The price and size of the CCD sensor reduce the practicability of such strip reader.

The CCD and the CMOS are highly popular image sensors [19, 24, 25]. The advantage of the CCD sensor lies on the stable image quality it produces. However, its complicated manufacturing process has largely limited its production to only a few companies, which led to the sensor’s high cost, especially for large-scale manufacture. By contrast, the CMOS is less expensive than the CCD of the same resolution. However, the quality of the image generated by CMOS falls short of that by CCD. Upon image capture, the CMOS sensor first converts the charge of every pixel to voltage and then amplifies the pixel before loading the image. The CMOS sensor can be driven by a power supply of 3.3 V, which corresponds to a lower power dissipation than that of the CCD. Another advantage of the CMOS sensor is that it can combine an analog-to-digital converter with a signal processor to produce a smaller CMOS sensor size than usual. Considering the abovementioned reasons, we chose the CMOS sensor over CCD as our image sensor.

### Image Processing Results

**Fig. 6:**
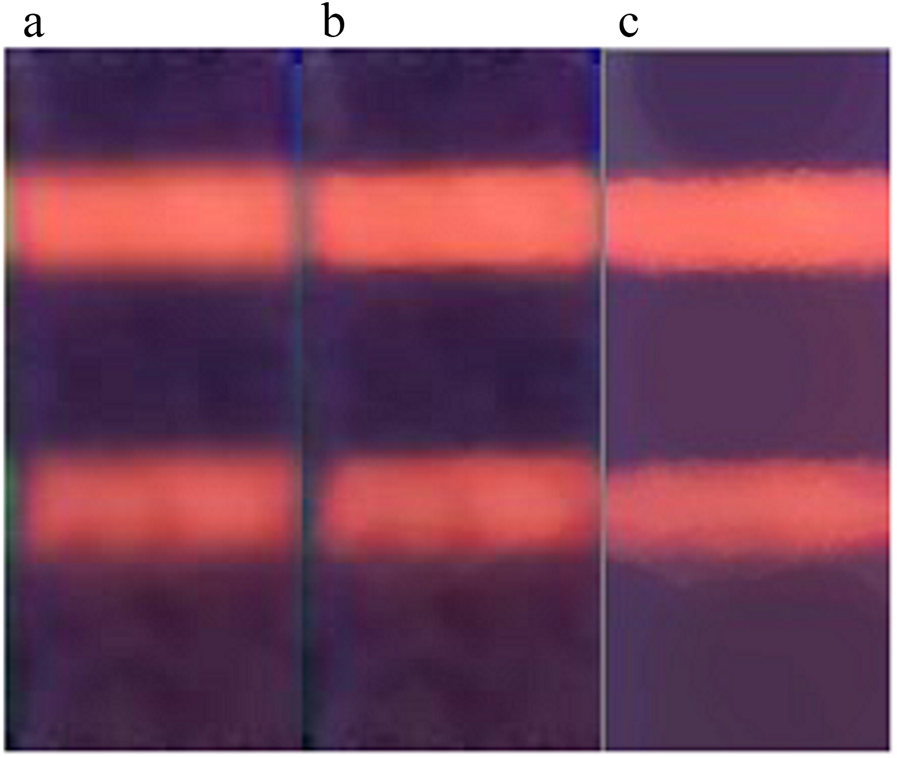
**a** Median filtering. **b** Gaussian filter. **c** Morphological filter

**Fig. 7:**
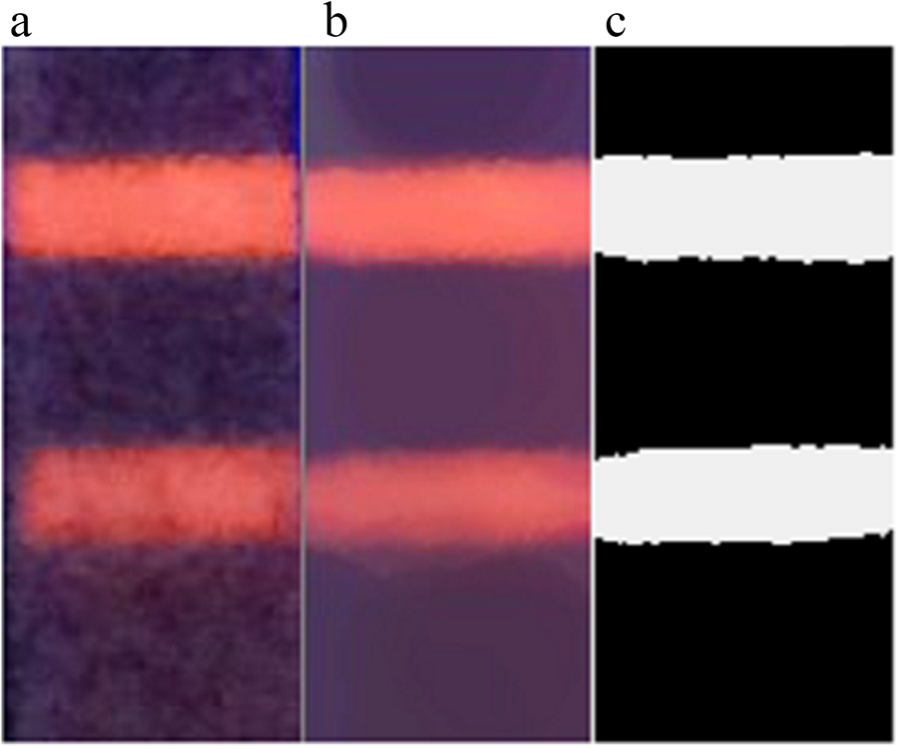
**a** Original image. **b** Image with morphological filter. **c** Image with Kmeans cluster algorithm

A raw image captured by the CMOS camera contained background noise (Fig. 7a). Several denoising algorithms were used to eliminate the background noise of the image (Fig. 6). As can be seen from the results, the morphological filter (Fig. 6c) performs better than the two other algorithms. After noise reduction, the Kmeans cluster algorithm was used to extract the C-line and the T-line (Fig. 7). Figure 6c shows that the colors of the C-line and the T-line were converted to white, whereas the other regions were converted to black.

**Fig. 8:**
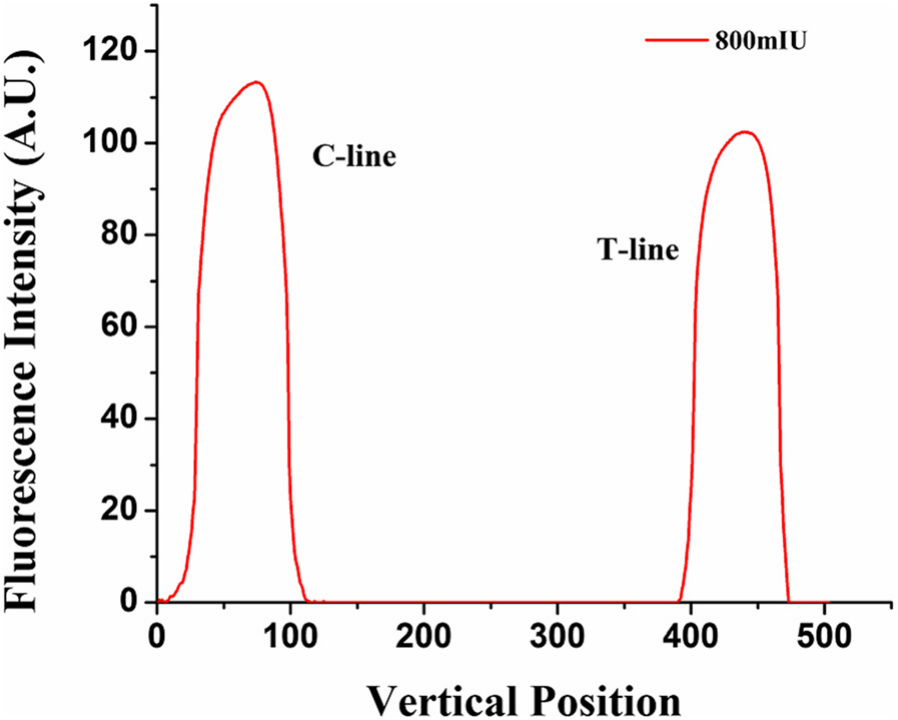
Fluorescence intensity of the test strip acquired by our reader acquired

Upon detection of the two lines, the reader calculates their average fluorescence intensities (Fig. 8). The fluorescence intensity of the C-line is higher than that of the T-line, which is consistent with the actual bio-reagent concentration differences between the two lines. This finding validates the effectiveness and accuracy of our algorithm.

### Repeatability and Reader Stability

**Fig. 9:**
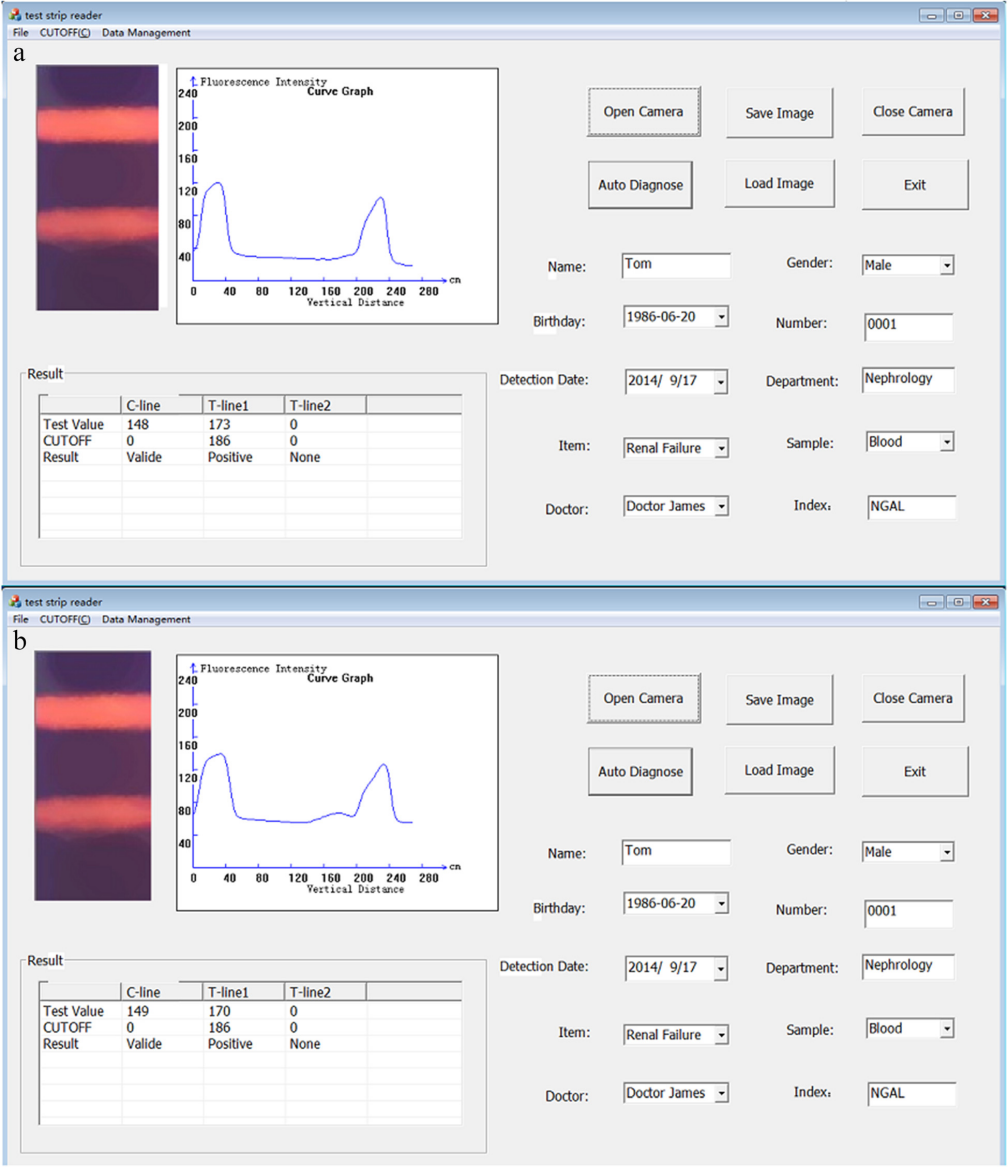
Test results of the same strip at different times. **a** and **b** are the detecting results of the different times about the same test strips.

**Fig. 10:**
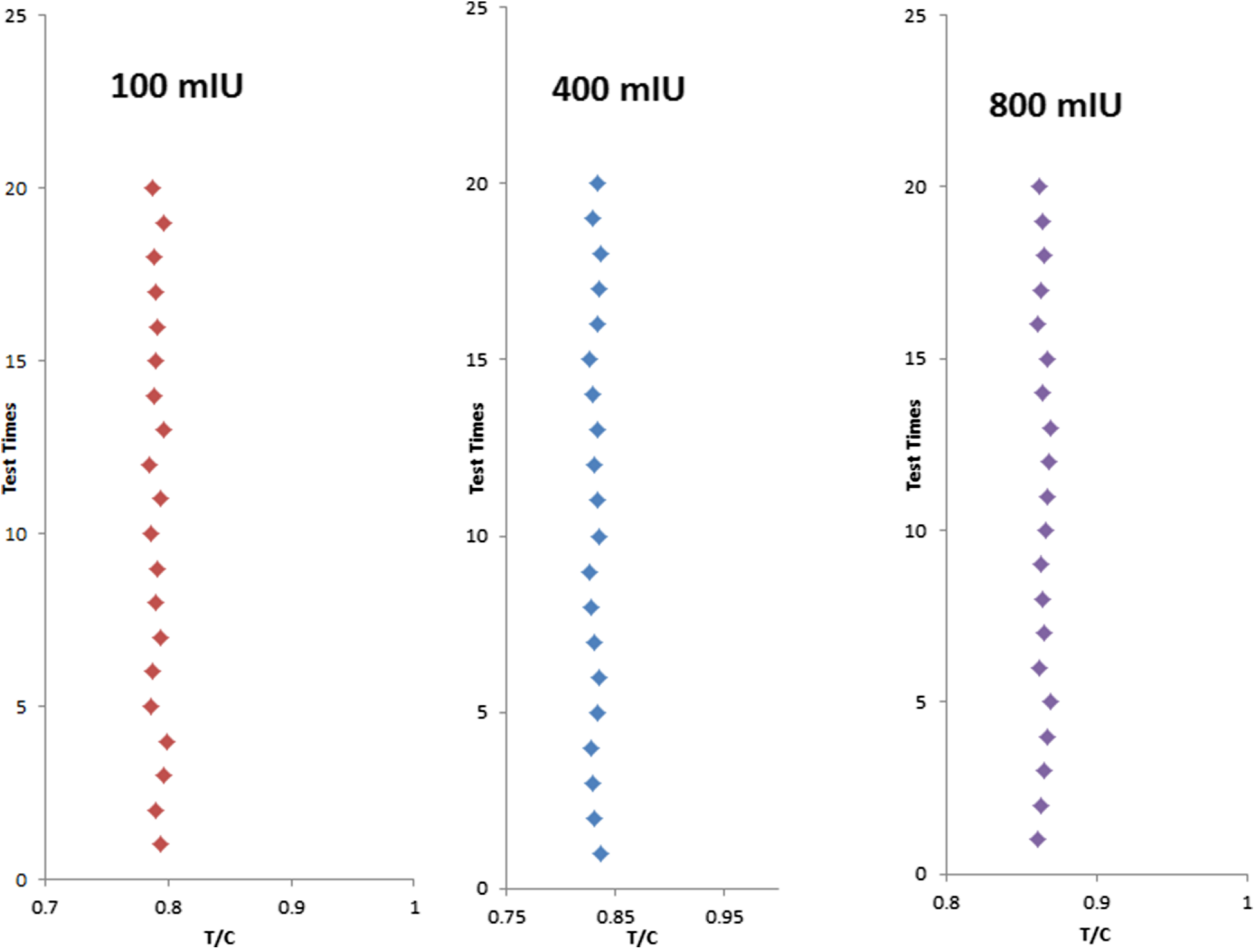
Ratios of T/C of three concentrations

To test the repeatability of our system’s readings, the same strip was inserted into our reader at different times. The changes in light intensity and strip location caused slight variations in the acquired image. This test was performed to detect the error tolerance of our reader, and the results are presented in Fig. 9. The values obtained at different times are nearly the same for each line, thus proving the good repeatability of our reader. To test reader stability, test strips containing three different concentrations (100, 400, and 800 mIU/mL) were used, and each concentration was used for 20 times of detection. Figure 10 shows the ratios of the C-line and T-line readings for the three concentrations. The discrepancies among these ratios were minimal, thereby verifying reader stability.

**Fig. 11:**
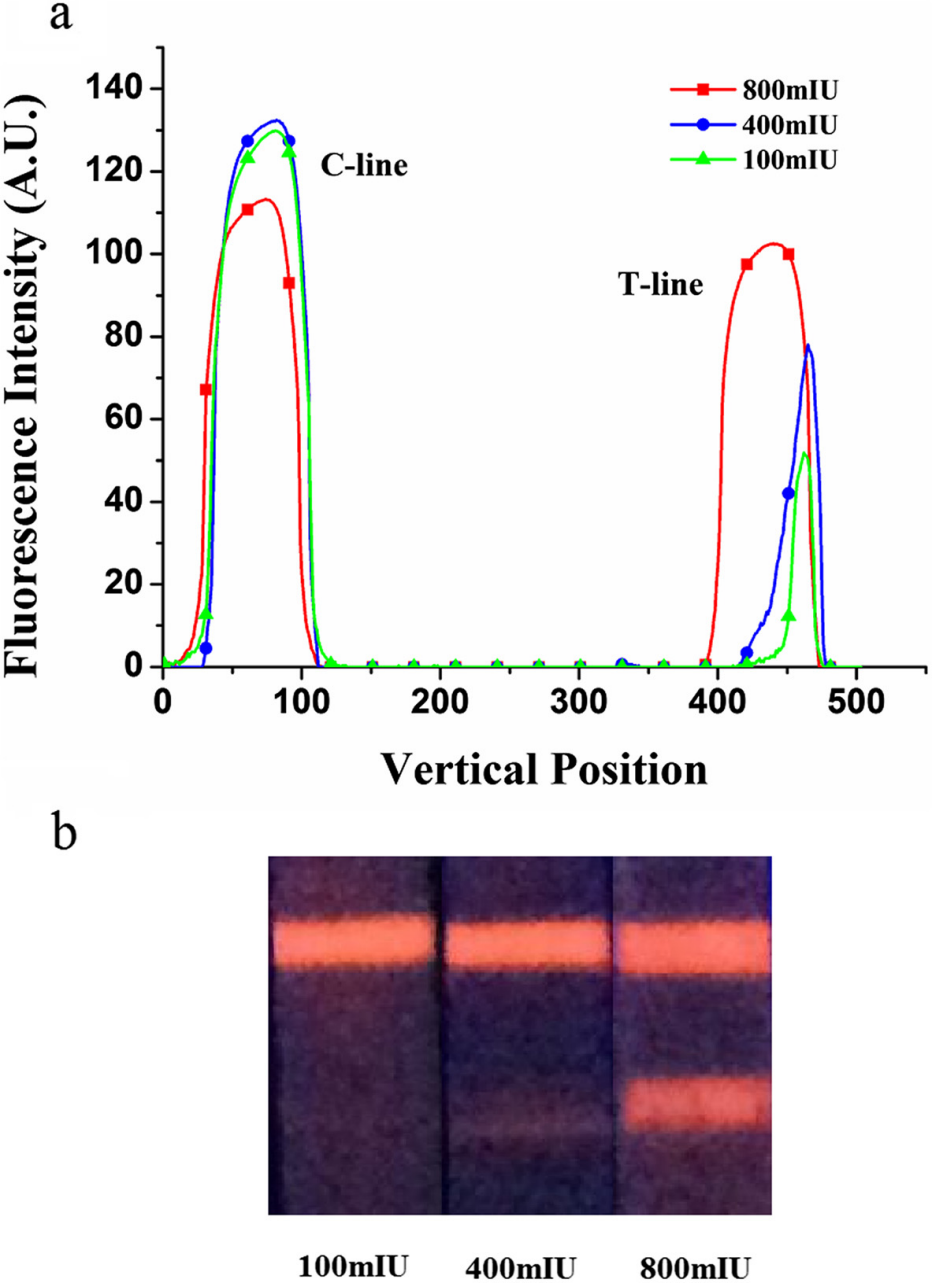
**a** Fluorescence intensity of three different concentrations. **b** Photos of strips of three different concentrations

To further confirm reader accuracy, different bio-reagent concentrations of the test strips were used. As shown in Fig. 10, the fluorescence intensities of the C-line and the T-line both grew with an increase in bio-reagent concentration. However, when the concentration reaches 800 mIU/mL, the fluorescence intensity of the C-line drops with an increase in bio-reagent concentration, whereas the fluorescence intensity of the T-line continues to increase (Fig. 11a). This phenomenon occurred because at exceedingly high bio-reagent concentrations, the antigen in the sample will adequately react with the antibody in the T-line, thus reducing the amount of the antigen that reaches the C-line. Consequently, the fluorescence intensity of the C-line drops with a reduction in antigen amount.

### Diagnosis of Urea-Enzymes Samples

To test the reader accuracy, 100 positive and 100 negative urea-enzyme samples were collected from a hospital. The detecting method hospital used was 13C-urea breath test, which had a high accuracy. All the samples were pretreated with the treating fluid which was prepared by our group’s members. The detection results are shown in Table 1. The specificity and sensitivity of the reader were 97 and 95 %, respectively, which were calculated according to the following formulas:

**Table 1:** Clinical test of urea-enzymes samples by using test reader

Sample size	Tested positive	Tested negative	Validity
100 positive	95	5	Sensitivity 95 %
100 negative	3	97	Specificity 97 %

$$ \mathrm{Specificity}=\frac{\mathrm{True}\ \mathrm{negative}}{\mathrm{True}\ \mathrm{negative}+\mathrm{False}\ \mathrm{positive}} $$

$$ \mathrm{Sensitivity}=\frac{\mathrm{True}\ \mathrm{positive}}{\mathrm{True}\ \mathrm{positive}+\mathrm{False}\ \mathrm{negative}} $$

To further validate the specificity, 100 positive cytotoxin-associated protein (CagA) samples were also collected to test the specificity of the reader. After the immunological reaction, only C-lines could be found in the NC membranes in all test strips by using the reader. Also, this experiment demonstrated that the detecting system had a high specificity.

**Fig. 12:**
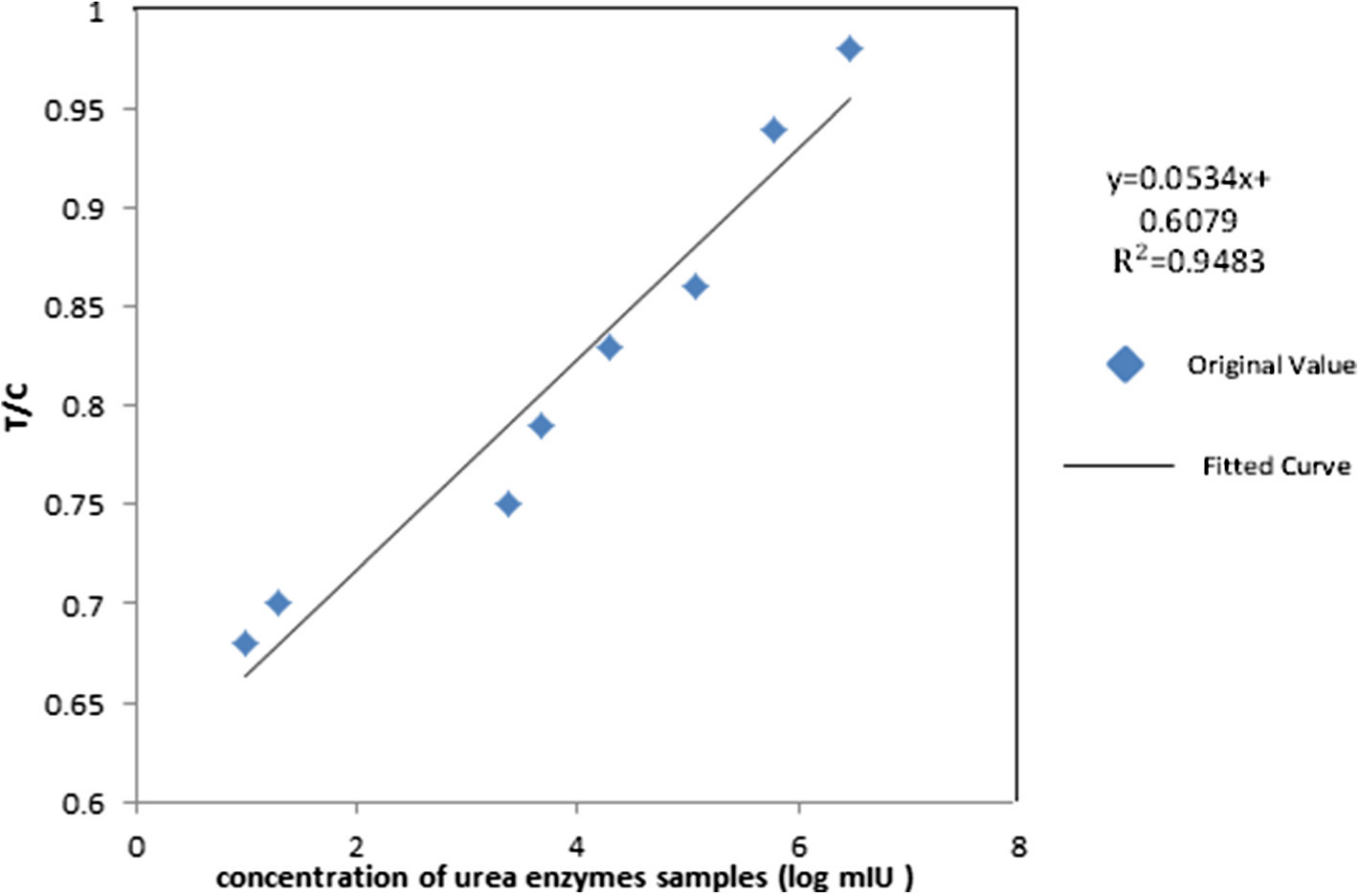
Graph of T/C in different concentrations

Furthermore, eight kinds of different concentrations of urea-enzyme samples were chosen, and each kind of concentration was subjected to detection by 20 different strips. The T/C value of every strip was tested, and the average value of the same concentration was calculated. The graphs of the original and fitted curves are shown in Fig. 12. With an increase in concentration, more antibodies are captured in the T-line, leading to an increase in the T/C ratio. The relationship between the T/C ratio and the concentrations can be illustrated by a fitting function. The detection limit of our reader was found to be 5 mIU/mL.

## Conclusions

A portable test strip reader that runs on a tablet PC and equipped with a Wi-Fi module was successfully developed in this study. This reader has a lighter weight than those of existing detection readers. It can run for long durations without an alternating current (AC) power supply, making it suitable for outdoor detection. Given the favorable price of the CMOS sensor, the cost of our reader is also tremendously low, making it an even practical device. The 3D printed cartridge of the LFIA is highly suitable for our reader, and 3D printing technology can be used to design different formats. The barcode printed on the strip contains the patient information and the detection type, thereby avoiding incorrect detections. The use of the barcode scanner enables the quick and accurate retrieval of information, which simplifies the operation.

In addition, a Kmeans clustering algorithm was used for image processing, and an application running on a tablet PC was designed to match the hardware system. Test strips of different concentrations were used to detect the stability and accuracy of our reader, which exhibited a good performance. The specificity and sensitivity of the reader were 97 and 95 %. The detection limit of the reader reached 5 mIU/mL. The results indicated that the system can quickly and accurately detect fluorescence signals. The system was also found to effectively improve detection quality. Its clinical performance demonstrated its suitability for point-of-care tests (POCTs). Overall, our reader can be valuable in the early diagnosis of several important diseases and help reduce morbidity worldwide.

## Abbreviations

AC: alternating current
ARM: Acorn Risc Machine
AutoCAD: Auto Computer-Aided Design
CCD: charge-coupled device
CMOS: complementary-metal-oxide-semiconductor
DAQ: data acquisition
HIS: hue-saturation-intensity
LED: light-emitting diode
LFIA: lateral flow immunochromatographic assay
PCs: personal computers
POCTs: point-of-care tests
PVC: polyvinyl chloride
RGB: red-green-blue
